# Shedding new light on the hidden complexity of seeds: chemically selective imaging of seed coats with stimulated Raman scattering microscopy

**DOI:** 10.1039/d4an01409j

**Published:** 2024-12-17

**Authors:** Chun-Chin Wang, Steven Penfield, Julian Moger

**Affiliations:** a Physics and Astronomy, University of Exeter Exeter EX4 4QL UK j.moger@exeter.ac.uk; b Department of Crop Genetics, John Innes Centre Norwich NR4 7UH UK

## Abstract

The seed coat plays a pivotal role in seed development and germination, acting as a protective barrier and mediating interac-tions with the external environment. Traditional histochemical techniques and analytical methods have provided valuable insights into seed coat composition and function. However, these methods often suffer from limitations such as indirect chemical signatures and lack of spatial resolution. Here, we introduce stimulated Raman scattering (SRS) microscopy as a novel analytical tool for non-destructive, label-free, high-resolution mapping of biopolymers, water and applied active ingre-dients (AIs) in intact seed coats. We demonstrate the capability of SRS microscopy to perform depth-resolved, chemically selective imaging of major seed coat biopolymers (pectin, tannin, and suberin). By comparing wild type arabidopsis thali-ana seeds with genetically modified mutants deficient in suberin and tannin, we illustrate the potential for semi-quantitative analysis of biopolymer content. Furthermore, we show that SRS microscopy can track the permeability of seed coats to wa-ter using deuterated water (D_2_O) uptake studies. Real-time imaging reveals differences in water permeation between wild type and suberin deficient seeds, highlighting the importance of seed coat composition in regulating water uptake during germina-tion. Additionally, we extend the application of SRS microscopy to large seeds, such as brassica oleracea, utilizing *epi*-detected imaging for surface studies. Finally, using a deuterated insecticide (clothianidin-d3), we demonstrate the capability of SRS microscopy to visualize the incorporation of AIs into seed coats. Our study presents SRS microscopy as a powerful tool for characterizing seed coat composition and understanding the diffusion of low molecular weight compounds into seeds. This technique offers new opportunities for designing seeds with tailored properties for improved germination and resilience to environmental stressors.

## Abstract 2

We introduce SRS microscopy as a novel analytical tool for non-destructive, label-free, high-resolution mapping of biopolymers, water and applied active ingredients (AIs) in seed coats.

## Graphical abstract

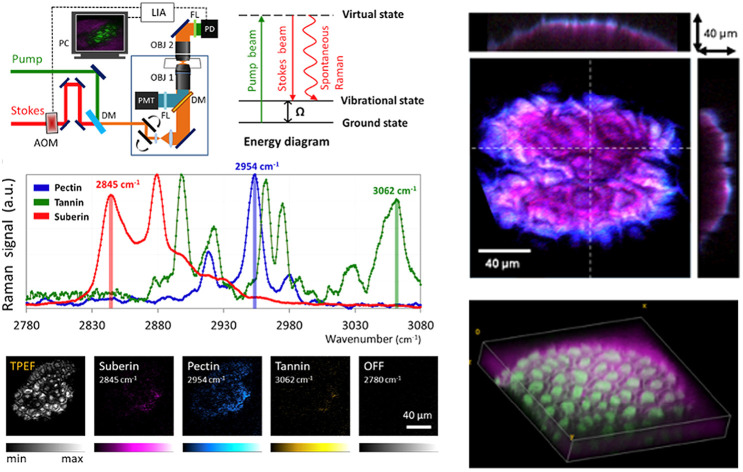

## Introduction

The seed coat is the outermost layer of seeds and serves as a dynamic interface between the developing embryo and the external environment. Initially considered a passive barrier, defending the embryo from external threats such as mechanical damage and UV radiation, the seed coat is now understood as a complex conduit for environmental cues that orchestrate critical processes during germination.^1,2^ Crop breeders strive to manipulate seed coat composition, either genetically *via* breeding or by chemical modification, to rationally engineer seeds that are optimised to germinate in specific environmental conditions and provide greater protection from pathogens.

Mapping the distribution of biopolymers within the seed coat, such as phenolics, and structural proteins provides crucial insights into seed coat structure and function. Gaining detailed knowledge of polymer distribution allows breeders to select seeds with coat properties that lead to uniform germination and growth; develop seeds that are resilient to environmental stressors; and track alleles affecting seed vigour and coat properties to facilitate targeted breeding efforts.^3^

Histochemical experiments have provided valuable insight into the seed coat by providing chemical analysis through the use of stains that give coloured, or florescent, products based on their chemical reactions with specific biopolymer components that can be spatially resolved using widefield or confocal microscopy.^4^ However, the resulting images are an indirect signature of the chemical composition that relies on chemical affinity of the stains for the compounds of interest and their ability to diffuse evenly through the sample. Moreover, distinguishing mixtures of polymers within the same region is problematic, and impossible to provide a quantitative comparison of relative polymer composition. In many cases, techniques require complex preparation that can yield inconsistent results unless performed under well controlled conditions. Furthermore, although stains have been used to investigate the permeability of the seed coat, they provide a measure of permeability defined by the chemical–physical properties of the dye and cannot be translated to the diffusion of physiologically relevant molecules, such as water, and low molecular weight agrochemicals.

Various label-free analytical techniques, including Fourier transform infrared (FTIR) spectroscopy,^5,6^ matrix-assisted laser desorption/ionization mass spectrometry imaging (MALDI-MSI),^7^ and magnetic resonance imaging (MRI)^8,9^ have been utilized to analyze seed coats. However, these methods lack spatial resolution to map the three-dimensional distribution of biopolymers within the seed coat, cannot be applied in intact seeds,^5–7^ and do not permit dynamic studies.^8,9^

Here we present stimulated Raman scattering (SRS) microscopy a as novel analytical method that overcomes the limitations described above to provides label-free, semi-quantitative, 3-dimensional high-resolution chemical-fingerprinting of multiple biopolymers in intact seed coats. SRS uses ultrafast laser pulses to drive a nonlinear light–matter interaction that enhances the signal from molecular vibrations to provide spatial-temporal mapping of molecular species based on the intensity of Raman bands. SRS generates a coherent nonlinear Raman signal by focusing pump and Stokes laser pulses into a sample with a difference in frequency matched to a Raman active mode of a molecular species of interest. In the presence of a Raman active mode, SRS results in a transfer of intensity between the two excitation beams that is detected by modulation transfer.^10^ This has previously been shown to give SRS a unique capability over other forms of coherent Raman scattering, such as CARS, for performing vibrational spectroscopy in strongly pigmented and fluorescent samples such as plant tissue.^11–16^ We demonstrate that SRS can perform depth-resolved chemically selective imaging of three major seed coat biopolymers, pectin, tannin and suberin, in intact wild-type arabidopsis thaliana seeds. We show that semi-quantitative comparison of biopolymer content of mutants deficient in suberin and tannin can be obtained. Time- and depth- resolved imaging using deuterated water (D_2_O) as a tracer for uptake into intact seeds is used to demonstrate that SRS can detect differences in the permeability of wild-type and suberin deficient seed coats. We show that SRS imaging can be performed in the *epi*-detection configuration to allow imaging of larger brassica oleracea seeds. Finally, using this method, we show that it is possible to image the deposition, and uptake, of a pesticide (clothianidin) into the seed coat.

## Results and discussion

**Fig. 1:**
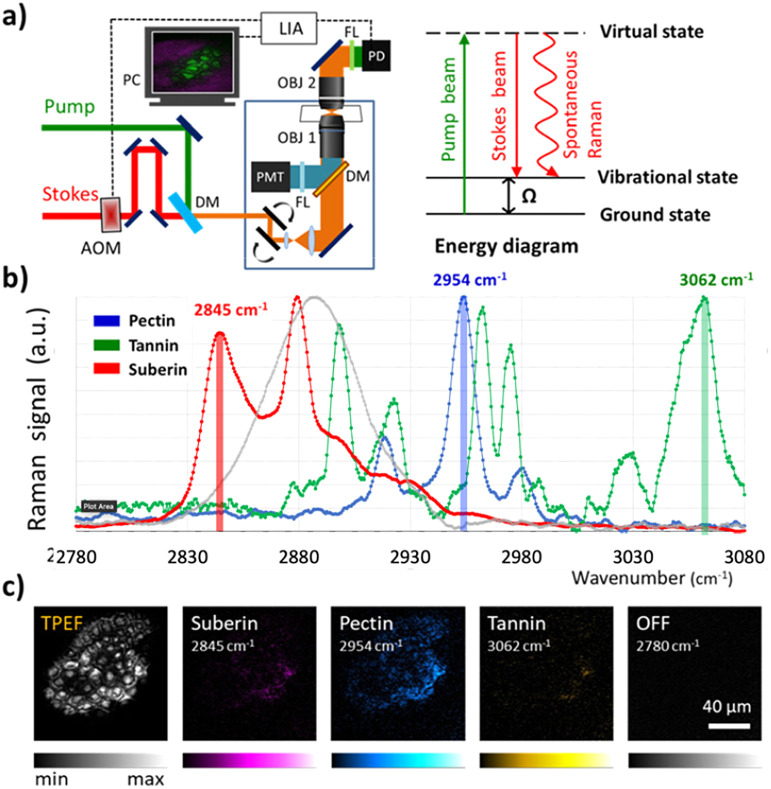
(a) Schematic of the optical setup and energy diagram. (b) Spontaneous Raman spectra of biopolymers (suberin, pectin, and tannin). (c) Two-photon excited fluorescence (TPEF) and SRS imaging of seed coat.

Raman spectra (Fig. 1b) of purified samples of pectin, tannin and suberin were acquired to identify intense isolated bands in the high wave number region (2780 cm^−1^ to 3080 cm^−1^) that could be used to provide chemically specific SRS contrast of the polymers. The long-chain fatty acid component of suberin provide a strong band at 2845 cm^−1^ corresponding to the abundance of the CH_2_ stretching mode. The characteristic CH_3_ carbohydrate band in pectin at 2954 cm^−1^ is distinct from the carbohydrate band in tannin, which exhibits a strong isolated band at 3062 cm^−1^ resulting from the aromatic CH stretch resulting from the polyphenolic structure. The peak assignments are consistent with previous Raman spectroscopy studies of pectin,^17–20^ suberin,^21^ and tannin.^22,23^ The Raman spectrum of cellulose (grey) illustrates that, although the CH stretch at 2897cm^−1^ overlaps with several bands of the three polymers under investigation in this work, it does not interfere with the peaks used for their chemically specific contrast.

Fig. 1c shows two-photon excited fluorescence (TPEF) and SRS images of suberin, pectin and tannin acquired using the Raman bands shown in 1b in the seed coat of an intact *Arabidopsis thaliana* seed. The mature seed coat is an external layer of dead cells which surrounds the endosperm and embryo. It consists of outer (oi) and inner (ii) integuments with distinct polymer components which serve to protect the embryo and to transmit information regarding the external environment. The outer integument contains two sub layers, the outermost, oi2, made up of hexagonal cells with thickened radial cell walls and a raised, volcano-like structure known as the columella. This layer contains a large quantity of a pectinaceous, complex polysaccharides. Below this is oi1, a brown pigmented layer (bpl) of pectinaceous polysaccharides and hydrophobic water proofing suberin and flavanols. The inner integument consists of palisade cells with walls rich with condensed polyphenolic tannins. A significant proportion of the biopolymers in both the outer and inner integuments exhibit autofluorescence, such as flavonols, tannin, proanthocyanidins, and suberin.^24^ Due to the overlap of spectral emission of these fluorophores, they do not provide chemical specificity, however, the ubiquitous nature of the signal provides a useful structural map of the seed coat which can be acquired in parallel with any one of the three biopolymers SRS signatures. The off-resonance image (2780 cm^−1^) validates the capability of SRS to selectively image the seed coat polymers against fluorescent background.

**Fig. 2:**
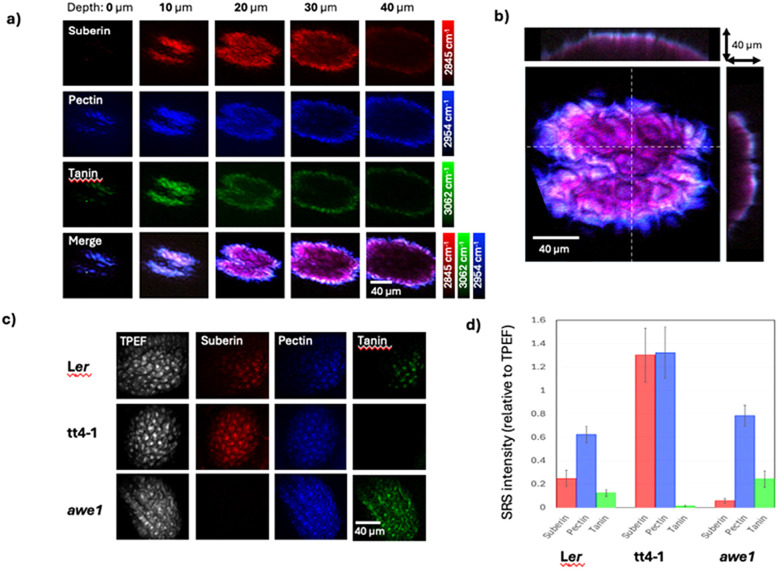
Chemically specific mapping of biopolymers in seed coats. (a) Depth-resolved imaging of biopolymers in wild-type arabidopsis seed. (b) Multiplanar reconstruction of color-merged images showing XZ and YZ slices. (c) Spatial distribution of biopolymers in wild-type (Ler) and gene-modified (tt4-1 and awe1) Arabidopsis seeds with SRS microscopy. (d) Relative SRS intensity of biopolymers (suberin, pectin, and tannin) in seed coats.

Fig. 2a shows depth-resolved SRS images of the three biopolymers, acquired using the Raman bands shown in 1b, in the seed coats of a wild-type (L*er*) *Arabidopsis* seed and the interaction and depth-dependent concentration of the polymers is highlighted in the colour merged image. A multiplanar reconstruction of colour-merged images is shown in 2b to provide visualisation of the depth dependent distribution of the three polymers. The SRS signal of pectin appears first, and then both suberin and tannin appeared at approximately 10 μm deeper than pectin from the seed surface. Moreover, the polyphenolic biomolecule (tannin) is mainly localized in the endothelium and parenchymatic layers in the testa of *Arabidopsis* seed. Beyond 20 μm into the seed coat the image is dominated by suberin. Our result shows that SRS imaging enables a clear demarcation between three major biomolecules based on the order of appearance of their respective signals. The results are consistent with those seen in histological experiments performed on cross-sections of *Arabidopsis* seeds.^25^

Fig. 2c presents the maximum intensity projection of TPEF and SRS images over a 40 μm depth in three different types of seeds (L*er*: wild-type; *tt4-1*: tannin-deficient; *awe1*: suberin-deficient). The TPEF is consistent between mutants and can therefore be used to normalise SRS intensity to account for depth-dependent signal attenuation. Fig. 2d compares the seed coat polymer composition, summed over the depth-normalised images, between the wild-type and the genetically modified *Arabidopsis thaliana* seeds. Error bars show the standard deviation of the depth-normalised pixel intensity for each polymer. The *awe1* mutant shows a 5-fold decrease in suberin content compared to the wildtype seed, which is consistent with previous studies made using gas chromatography mass spectroscopy.^26^ The absence of tannin in *tt4-1* has previously been confirmed using light microscopy of seed coat sections.^27^ The accumulation of both tannin and suberin in the seed coat play a role in controlling dormancy,^28^ and in the case of tt4-1 the tannin deficiency is compensated with an increase in suberin. Although these observations have previously been made with conventional analytical techniques, this is the first demonstration of label-free, non-destructive, imaging of these polymers in intact seeds, simultaneously.

**Fig. 3:**
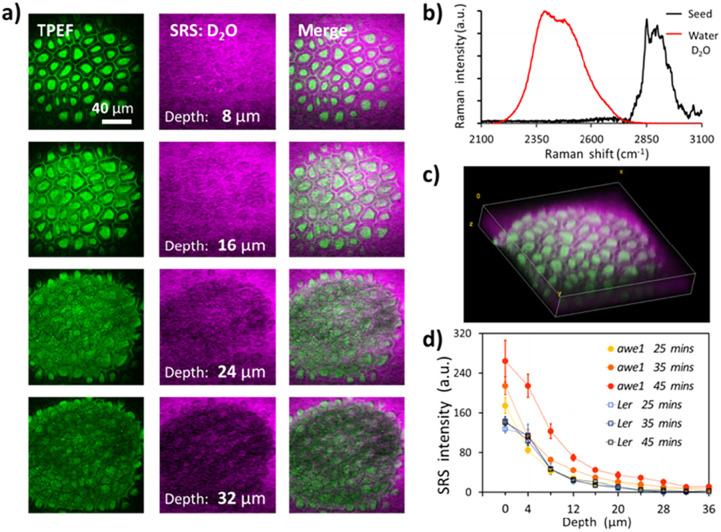
Water uptake in arabidopsis seeds. (a) Depth-resolved imaging of two-photon excited fluorescence (TPEF) and heavy water (D_2_O) in wild-type arabidopsis seed. (b) Spontaneous Raman spectra of D_2_O and arabidopsis seed. (c) 3D reconstruction of water and seed biopolymer distribution. (d) Time-lapse and depth-resolved water uptake in wild-type (Ler) and gene-modified (awe1) arabidopsis seed coats.

Fig. 3a shows the depth-resolved SRS images of an *Arabidopsis* seed following 45 min imbibition in deuterated water. As shown in Fig. 3b, SRS of the O–D stretch (2500 cm^−1^) provides a unique chemical signature of the imbibed water than can be distinguished from endogenous O–H content. TFEP provides a useful structural reference of the seed coat, which is used to define the seed coat surface, labelled as 0 microns, as the plane that intersects the columellae and the surrounding cell walls. After 45 min the D_2_O signal can be seen up to a depth of 24 microns into the seedcoat, reaching the inner integuments (ii). Fig. 3c shows a 3D projection of the depth-resolved images. The columellae and the cell walls remnant structures of wild-type *Arabidopsis* seed remain roughly the same after 45 min of water imbibition, suggesting that the low permeability of wild-type L*er* seed.

The seed coat has been shown to control germination by regulating permeability to water.^29^ Suberin is thought to play a key role and seeds deficient in suberin show increased permeability to applied tetrazolium dyes.^2,30^ While dyes provide a useful measure of seed coat permeability, their ability to permeate the seed coat does not present that of water. Fig. 3d compares time- and depth-resolved D_2_O content averaged over each image frame and normalized against the TPEF signature to account for depth-dependent signal attenuation, inside the seed coats of wild-type (L*er*) and suberin deficient (*awe1*) seeds. After 25 min of imbibition, *i.e.* the first time point, both L*er* and *awe1* seeds show similar concentration profiles, water penetrating to a maximum depth of 24 μm. It should be noted that 0 μm is defined by the first image in the stack that contains TPEF from the seed coat and therefore at the first time point the D_2_O signal from the *awe1* seed is slightly higher than L*er* due to the diffusion that has taken place in the first 25 min. However, at 35 and 45 min time-points the profiles diverge and the *awe1* seeds show a significantly higher uptake and reaching maximum depth of 36 microns, while the L*er* profile remains unchanged.^31^

**Fig. 4:**
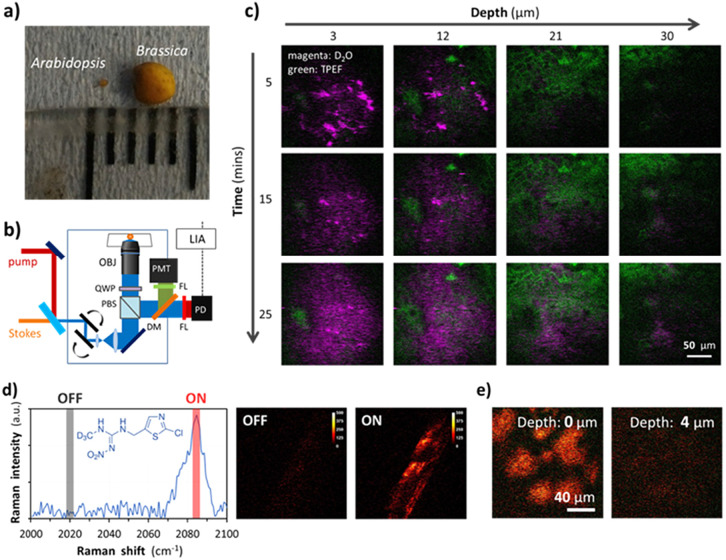
Epi-SRS of brassica seeds. (a) Compares the size of Arabidopsis thaliana and brassica oleracea seeds. (b) Schematic of the *epi*-detection setup. (c) SRS microscopy visualising D_2_O uptake into the intact seed coat of Brassica oleracea. (d) Spontaneous Raman spectrum and chemical structure of the deuterated insecticide, clothianidin-d_3_. (e) *epi*-SRS imaging of clothianidin-d_3_ on brassica seed coat.

*Arabidopsis* seeds provides a good model to explore the effects of mutants on the seed coat, however they are not representative of the majority of crop plants which are the focus of studies of germination vigour. Fig. 4a compares the size of *Arabidopsis thaliana* and *Brassica oleracea*, an example cruciferous vegetable showing that *Brassica* seeds are significantly larger than *Arabidopsis* seeds. SRS is inherently phase locked with one of the incident beams which propagate in the forward direction. For thin samples, such as the *Arabidopsis thaliana* seeds, SRS can be detected in forward-propagating ballistic photons. However, due to the size and opacity of *Brassica oleracea* seeds, forward detection (transmission) imaging is not possible and *epi*-detection becomes the only choice. As illustrated in Fig. 4b, *epi*-detected SRS imaging was performed with the introduction of a quarter-wave plate and polarizing beam splitter and the (weaker) back-scattered SRS signal pre-amplified before lock-in detection. Fig. 4c presents the time-lapse 3D imaging of D_2_O uptake into the intact seed coat of *Brassica oleracea* with dual-modal microscopy. The permeation of D_2_O into seed can be monitored as a function of seed coat hydration to allow quantification of diffusion.

This capability can be expanded to allow chemically specific mapping of applied chemical agents, such as agrochemical insecticide formulations. Fig. 4d shows that Clothianidin-d_3_ contains a C–D_3_ bond which provides a unique Raman peak, that appears in the “silent region” (2000–2800 cm^−1^) of the spectrum where there is no interference from endogenous seed components. The ‘ON’ (2083 cm^−1^) and ‘OFF’ (2020 cm^−1^) resonance images confirm the ability to selectively image the active ingredient (Clothianidin-d_3_). Finally, we acquired *epi*-SRS on-resonance (2083 cm^−1^) imaging of Clothianidin-d_3_ on the surface of an intact *Brassica* seed. The *epi*-SRS images at different depths of seed coat showed the permeation of clothianidin-d_3_ into *Brassica* seed coat during the first five minutes after uptake of the insecticide, as shown in Fig. 4e.

The variation in the environmental temperature during seed production causes a large variation in seedling growth vigour after germination, and this difference in vigour is related to the tissues surrounding the embryo in the seed, including the seed coat and endosperm structures. In *Arabidopsis thaliana* seeds, changes in tannin content caused by temperature effects are associated with altered seed coat permeability to water,^2^ therefore in the future our imaging platform can be applied to examine the mechanism through which temperature variation affects *Arabidopsis thaliana* seed coat permeability.

The results presented represent the first exploration of the capabilities of SRS to selectively image seedcoat polymers. We have shown that a single-band SRS approach provides chemically specific contrast of pectin, tannin and suberin. However, with more advanced SRS imaging approaches, such as spectral-focusing hyperspectral SRS,^31^ it may be possible to detect more components simultaneously, such as cellulose or, for example, to explore lignin production in arabidopsis seed coats under cold conditions.^32^ Moreover, it would be possible to image biopolymers and AIs simultaneously on the surface of seeds by implementing two-colour or multi-colour imaging techniques.^11,33–37^Moreover, the penetration depth of label-free imaging could be extended using longer wavelength excitation,^38^ which might allow deeper tissue imaging during seed germination and plant development.^21,39,40^ Finally, deuterium oxide (D_2_O) could be used as a universal probe to track *in situ* metabolic activities through the emergence of C–D bond-containing macromolecules. Combing D_2_O probing with SRS microscopy will enable us to image the biosynthetic incorporation of deuterium from D_2_O into biomolecules in living plant seed development.^41,42^ While previous studies have been able to measure water uptake into seeds with micro-MRI^43^ in larger seeds (*i.e.*, soya), this is first demonstration of high-resolution, water imaging in the first few layers of the seed coat and the first visualisation of water moving around the columellae. This capability could enable future studies to obtain values of permeability of this important interfacial layer.

## Experimental

### Stimulated Raman scattering (SRS) microscopy

Microscopy was performed using a custom-built imaging platform based on a laser scanning microscope and an ultrafast laser system. A schematic of the optical setup is shown in Fig. 1a. The laser source consisted of a picosecond mode-locked fibre laser (aeroPULSE, NKT Photonics) providing 2 ps pulses at 1031 nm and 76 MHz repetition rate, which was frequency-doubled to pump an optical parametric oscillator (OPO, Levante Emerald, APE) to provide a signal beam tuneable from 690 to 990 nm, which served as the pump beam. The fundamental 1031 nm from the fibre laser served as the Stokes beam and was modulated at 8.4 MHz using an acoustic optical modulator (MT200-A0.5-1064, AA Optoelectronic). Imaging was carried out on a modified confocal laser scan unit (FluoView 300, Olympus) and an inverted microscope frame (IX71, Olympus). The pump and Stokes were spatially and temporally overlapped before being focusing onto the sample using a 60× NA1.2 water immersion microscope objective (UPLSAPO60XW, Olympus). The pump and Stokes laser powers on the sample were 6 and 12 mW, respectively.

The forward propagating SRS signal, used to image the *Arabidopsis thaliana* seeds, was collected using a 60× NA1.0 water immersion microscope objective (LUMPLFLN60XW, Olympus). The Stokes beam was blocked with a band-pass filter (890/220 nm, Chroma) and the pump beam detected using a Si photodiode (FDS 1010, Thorlabs) with a 70-volt reverse bias. A low-pass filter (BLP-21.4+, Mini-Circuits) was used to reject the 76 MHz signal from the laser repetition-rate and the SRS modulation transfer signal detected at 8.4 MHz on the X-channel of a lock-in amplifier (SR844, Stanford Research Systems). A 20 μs integration time was used resulting in a 5.3 seconds frame rate of 512 × 512 pixels images.

Two-photon excited fluorescence (TPEF) was detected simultaneously in the *epi*-direction using a 750 nm long-pass dichroic mirror (750dcxr, Chroma) and two filters centred at 660 nm (660.0 IF 40D, Ealing) to isolate the fluorescent emission before detection on a photomultiplier tube (PMT, R3896, Hamamatsu). Image acquisition and processing were performed using ScanImage (Vidrio Technologies) and open-source software Fiji (National Institute of Health, NIH), respectively.

To image the *Brassica oleracea* seeds with *epi*-detection, a quarter-wave plate (AQWP10M-980, Thorlabs) and polarizing beam splitter (CCM1-PBS252, Thorlabs) were used to collect the back-scattered SRS signal. The backscattered Stokes signal was blocked with a band-pass filter (890/220 nm, Chroma), and the pump beam were detected by an amplified Si photodiode (DET36A, Thorlabs), and detected by a LIA in a similar manor to the forward SRS signal. The schematic of the *epi*-SRS setup is shown in Fig. 4b.

Pump and Stokes beams with frequencies *ω*_p_ and *ω*_S_ are incident upon the sample with the frequency difference *ω*_p_ − *ω*_S_ has chosen to match the molecular vibrational frequency (Ω) of interest. For the SRS, spectral acquisition regions of interest were acquired as the OPO was sequentially tuned to provide pump wavelengths, giving values of *ω*_p_ −*ω*_S_ over the spectral range 2010–3080 cm^−1^.

### Raman spectroscopy

Raman spectra of plant biopolymers (purified pectin, suberin, and tannin), heavy water (deuterium oxide, D_2_O), and deuterated insecticide (clothianidin-d_3_) were acquired using a Renishaw RM1000 Raman microscope (Renishaw) equipped with a 1200 lines per mm grating providing a spectral resolution of 1 cm^−1^ and a diode laser providing excitation at 785 nm with up to 300 mW power. The system was calibrated prior to every spectral acquisition using the Raman band of a silicon wafer at 521 cm^−1^. Spectral data were acquired using Renishaw v.1.2 WiRE software, baseline subtracted and corrected for the intensity response of the CCD detector.

### Seed samples

All seeds used in the study were produced at the John Innes Centre (Norwich, UK). The variety Landsberg erecta (Ler) were used to represent the wild type arabidopsis thaliana seed. A tannin deficient mutant, transparent testa (tt4-1), and a suberin deficient mutant, awake1 (awe1), were used to demonstrate the ability of our method to quantify polymer content and to investigate the effect of the suberin deficiency on seed coat permeability. Brassica oleracea seeds were used to demonstrate the capability of the enface, *epi*-detection to image large seeds.

All seeds were imaged intact, *i.e.* without sectioning or cutting. Arabidopsis thaliana seeds were mounted between two glass coverslips and held in place using self-adhesive slide spacers to prevent movement during imaging. Brassica oleracea seeds were adhered to a single glass coverslip had help in place by self-adhesive spacers. All reagents, reference seed coat polymers, D_2_O and Clothianidin-d3 were supplied by Sigma Aldrich.

## Conclusions

In Summary, we have demonstrated, for the first time, that SRS microscopy is a powerful analytical tool for investigating the intricate composition and dynamics of seed coats. SRS overcomes the limitations of existing techniques to provide label-free, semi-quantitative, high-resolution chemical-fingerprinting of the key biopolymers within intact seed coats. Our results showcased the potential of SRS microscopy for quantitative analysis, as evidenced by the comparison of biopolymer content between wild-type and genetically modified seeds.

The ability to studying seed coat permeability by tracking the uptake of deuterated water into intact seeds represents a significant advance in analytical capability. Our results show that dynamic imaging reveals differences in water penetration between wild-type and suberin-deficient seeds, confirming the role of suberin in controlling seed coat permeability.

We successfully applied *epi*-SRS to image to large crop seeds such as brassica oleracea, demonstrating the commercial applicability of the technique. Finally, we showcased the ability of SRS for studying the uptake of agrochemicals into seed coats, paving the way for future investigations into pesticide interactions and seed treatment efficacy.

Looking ahead, SRS microscopy holds promise for advancing our understanding of seed coat biology and enhancing crop breeding efforts. By enabling 3D imaging of multiple biopolymers and agrochemicals, SRS microscopy opens up new avenues for exploring seed development and environmental responses, such as monitoring chemical changes during germination in different environmental conditions. Moreover, due to the lack of sample preparation required, in principle, SRS imaging could be automated to screen large numbers of seed alleles to quantity seedcoat polymer content. Overall, our study establishes the value of SRS microscopy as a transformative tool for unravelling the complexities of seed coat composition and function.

## Author contributions

The manuscript was written through contributions of all authors. All authors have given approval to the final version of the manuscript.

## Data availability

The data that support the findings of this study are available from the corresponding author upon reasonable request.

## Conflicts of interest

The authors declare no competing financial interest.
